# Systems Biology Approach for Cancer Vaccine Development and Evaluation

**DOI:** 10.3390/vaccines3030544

**Published:** 2015-07-14

**Authors:** Luisa Circelli, Annacarmen Petrizzo, Maria Tagliamonte, Maria Lina Tornesello, Franco M. Buonaguro, Luigi Buonaguro

**Affiliations:** Laboratory of Molecular Biology and Viral Oncology, Department of Experimental Oncology, Istituto Nazionale per lo Studio e la Cura dei Tumori, “Fondazione Pascale” - IRCCS, Naples 80131, Italy; E-Mails: lulacir@libero.it (L.C.); annacarmen.petrizzo@tiscali.it (A.P.); m.tagliamonte@istitutotumori.na.it (M.T.); m.tornesello@istitutotumori.na.it (M.L.T.); irccsvir@unina.it (F.M.B.)

**Keywords:** systems vaccinology, cancer vaccine, cancer immunotherapy

## Abstract

Therapeutic cancer vaccines do not hold promise yet as an effective anti-cancer treatment. Lack of efficacy or poor clinical outcomes are due to several antigenic and immunological aspects that need to be addressed in order to reverse such trends and significantly improve cancer vaccines’ efficacy. The newly developed high throughput technologies and computational tools are instrumental to this aim allowing the identification of more specific antigens and the comprehensive analysis of the innate and adaptive immunities. Here, we review the potentiality of systems biology in providing novel insights in the mechanisms of the action of vaccines to improve their design and effectiveness.

## 1. Introduction

Vaccines are the most powerful measures to prevent the burden of infectious diseases and represent the greatest success in the history of public health, especially for microbial pathogens that are antigenically stable and unable to evade the host immune response [1,2]. Indeed, vaccines play a great role in diminishing mortality and morbidity from major global infections [3].

The goal of a successful vaccine is to induce long-term protective immunity based on the generation of an antigen-specific immunological memory. This is achieved via several levels of cross-talks between the innate and adaptive immune systems, involving both cell to cell contact and/or soluble factors (*i.e*, cytokines and chemokines). Most of the current successful vaccines are based on live attenuated or inactivated pathogens that show immunological specific characteristics. The live attenuated vaccines are viruses with a limited replication in the vaccinated host, mimicking a natural infection and spread to multiple host immune organs or tissues as well as eliciting immune responses similar to those induced by fully-replicative pathogens. On the contrary, the inactivated vaccines do not replicate and are safer than live attenuated vaccines. However, they are generally less effective, requiring multiple administrations to boost the antibody titer over time [4]. In the last years, recent advances in genomics and proteomics have provided essential tools to develop alternative non-replicating vaccine strategy, including recombinant proteins, synthetic peptides, DNA, and particulate structures (*i.e*, virus-like particles) [5,6]. In this regard, the holistic approach applied to vaccinology from discovery of new antigens to evaluation of vaccine efficacy, is defined *systems vaccinology*.

## 2. System Vaccinology

The conventional immunological methods, (*i.e*, ELISA, ELISPOT, flow cytometry), used to assess efficacy of vaccines have played a valuable role in the field of vaccinology and will remain essential in evaluating responses to vaccination in the future. However, such approaches are generally only able to analyze a single or small number of components of the immune system at a given time, and are insufficient to analyze the full complexity of the structure and dynamics of the human immune system as a whole. This represents a critical obstacle towards understanding the molecular mechanisms by which vaccines generate protective immune responses and identifying meaningful correlates of protection. By examining how coordinated interactions at a molecular level give rise to immune responses, systems biology approaches enable a holistic view of the immune system and its many components. This developing field provides many promising omics’ tools (*i.e*, genomics, transcriptomics, proteomics, metabolomics) to overcome the challenges facing current vaccine development.

Advances in high-throughput technologies as well as significant reduction of costs have granted researchers the ability to interrogate the properties and abundances of entire classes of molecular components within the immune cells [7,8]. Simultaneously, analytical chemistry techniques have been potentiated to identify and quantify metabolites at cellular as well as tissue level for the so-called *metabolomics* [9], allowing identification of metabolic activities associated with immune responses [10] as well as inflammation [11].

**Figure 1:**
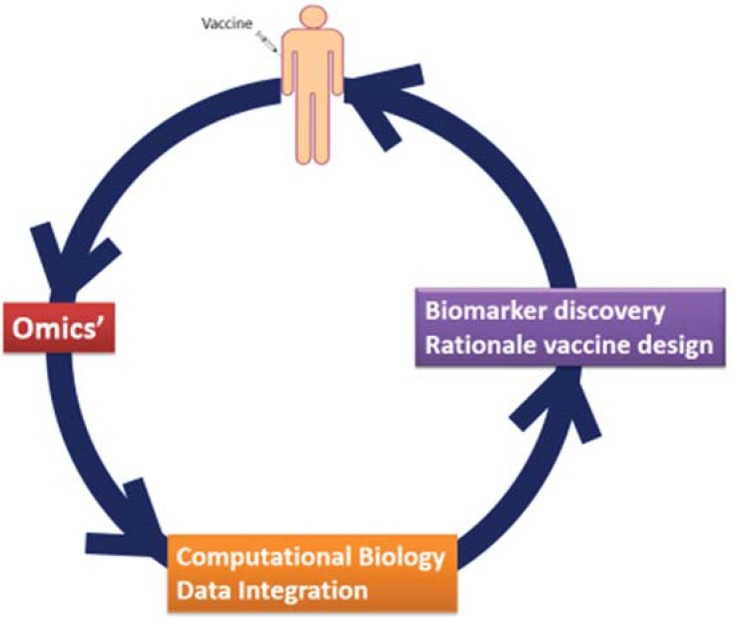
Systems biology approaches for vaccine studies interactions and the implications on translational research.

Systems vaccinology is an emerging field that applies such “omics” technologies to study immune responses to vaccination in combination with bioinformatics tools such as transcriptional network analysis and predictive modeling [12,13,14,15]. As a systems-based approach, it aims to integrate data generated by highthroughput measurements in the context of vaccination, in order to characterize the interactions between individual components of the immune system in pursuance of understanding and predicting behavior of the system as whole. This includes analysis of transcriptional, signaling, and metabolic pathways whose activities are perturbed in the various cells of the immune system in response to vaccination, as well as identification of molecular signatures that are predictive of protection from infection (Figure 1). The knowledge obtained through such analyses can support the rational design of new vaccines to generate long-lasting protection and induce improved responses in different target groups, including subjects with diminished immune function such as immunocompromised patients and the elderly [16].

## 3. System Vaccinology for Infectious Disease Vaccines

The first examples of the use of gene transcriptional profiling to evaluate immune responses to vaccination were performed on the yellow fever vaccine, a live-attenuated vaccine (YF-17D) that induces potent and long-lived CD8+ T cell and neutralizing antibody responses [17,18]. Following these initial studies, systems biology approaches have been used to examine immune responses to vaccines against a wide range of pathogens, including influenza [19,20], malaria [21] and HIV [22,23,24].

Different vaccine antigens or adjuvant systems are likely to induce different innate and adaptive responses, making extrapolation from different trials challenging. Indeed, significant differences in genes responding to a protein subunit influenza vaccine versus a conjugate polysaccharide pneumococcal vaccine have been reported [25], likewise differences have also been described between live and non-living vaccines [26].

In parallel, candidate gene studies as well as genome-wide association studies are used to identify polymorphisms in genes associated with improved or diminished vaccine responses [27].

Associations between the same single nucleotide polymorphisms (SNPs) in the TLR3 gene promoter and the downstream intracellular signaling molecules with humoral and cellular responses have been described for measles vaccination [28] and rubella vaccination [29]. Alternatively, specific SNPs in the promoter region of the TLR4 gene as well as haplotype-tagging SNPs in genes of the TLR signaling pathway have been shown to influence the antibody response to pertussis vaccine [30,31].

Furthermore, the association of human leukocyte antigen (HLA) variations with the responses to single vaccines has been investigated for different vaccines and recently reviewed [32]. Interestingly, four HLA alleles, such as DRB1*07, DQA1*02:01, DQB1*02:01, and DQB1*03:03, seem to be significantly associated with the absence of antibody response to the measles, mumps, and rubella (MMR-II), Hepatitis B virus (HepB), or influenza vaccines, whereas two HLA alleles, such as DRB1*13 and DRB1*13:01, seem to be significantly associated with positive antibody responses to the MMR-II, HepB, or influenza vaccines.

## 4. Biosignatures for Vaccine Safety and Efficacy

Systems vaccinology approaches can be extremely effective to identify biosignatures of safety and efficacy in pre-clinical studies, to prioritize available candidates, and in early clinical development, to avoid later failure. Indeed, late stage clinical vaccine trials have unexpectedly shown lack of efficacy either completely [33,34] or partially [35], or raised safety concerns [33].

Parameters may be identified retrospectively but post hoc analyses may identify misleading chance correlations. An alternative approach is to use vaccines in translational studies to dissect out mechanisms of reactogenicity and efficacy, and such an approach was taken in the MRKAd5/HIV vaccine efficacy trial [36]. Indeed, within 24 h a striking increase in peripheral blood mononuclear cell gene expression associated with inflammation, interferon (IFN) response, and myeloid cell trafficking occurred. However, such responses were strongly attenuated in vaccinees with preexisting adenovirus serotype 5 (Ad5) neutralizing antibodies, suggesting that Ad5-seropositive subgroups may have suffered from the lack of appropriate innate activation. Moreover, patterns of chemoattractant cytokine responses at 24 h and alterations in 209 peripheral blood mononuclear cell transcripts at 72 h were predictive of subsequent induction and magnitude of HIV-specific CD8+ T-cell responses. Such results strongly support the evidence that systems vaccinology may allow selection of vaccine candidates eliciting innate immune response profiles more likely to drive protective immunity.

## 5. Systems Vaccinology for Cancer Vaccines Application

**Figure 2:**
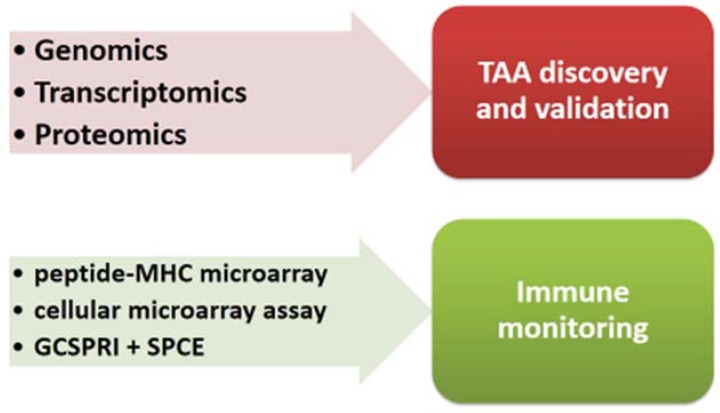
Systems vaccinology approach applied to cancer vaccines.

Systems vaccinology is applied to cancer vaccines also for identification of specific shared or personalized TAAs and for the immunomonitoring of cellular immunity elicited in vaccinated subjects (Figure 2).

### 5.1. Identification of Tumor Associated Antigens (TAAs)

TAAs derive from cellular proteins and should be mainly or selectively expressed on cancer cells to avoid either immune tolerance [37,38] or autoimmunity effects [39]. Integration of different high-throughput strategies allows for the identifation of cancer-specific proteins from which specific TAAs can be derived. Indeed, parallel examination of the genome (genomics), transcriptome (transcriptomics), proteome (proteomics) and, more recently, metabolome (metabolomics) in tumor samples, compared with normal samples, allows for integration of the relationships of several and theoretically all the elements in a system [40]. Nevertheless, a multilevel evaluation of a tumor has been applied in a limited number of studies, showing that in contrast to relatively high number of genes differentially expressed between normal and tumor tissues, very limited number of proteins correlated with the transcriptional changes [41,42]. Such results clearly show that only the integration of data looking at different steps of the system is able to provide more accurate information on the differential protein expression in tumor cells.

Once tumor-specific proteins are identified, relevant immunogenic major histocompatibility complex (MHC) class I peptides can be predicted by computational algorithms or immunoinformatics technologies [43,44] and then tested experimentally for immunogenicity (e.g., ELISPOT, Tetramer binding assay).

More recently, strategies based on high resolution mass spectrometry (MS) have been developed for analyzing the *HLA ligandome* on tumor cells, to identify naturally processed class I and class II tumor-associated peptides [45]. This strategy, indeed, allows for the identification of T cell epitopes in fact presented by the tumor cells, thus representing a valid target of the T cells, and it has been employed to identify the HLA ligandome for glioblastoma (GB) [46], renal cell cancer (RCC) and colorectal cancer (CRC) (reviewed in [47]).

### 5.2. Identification of Tumor Associated Antigens (Mutanome)

In the quest of the most specific tumor-associated antigens, next-generation sequencing and computation prediction allow for the identification of genetic alterations in cancer cells of each cancer patient (the mutanome) encoding unique mutated peptides (m-peptides) that can be used as vaccines to elicit specific anti-tumor T cells [48,49]. Indeed, cancer genome instability and subsequent selective pressure lead to accumulation of mutations that may give rise to non-synonymous nucleotide substitutions which may lead to the expression and presentation of mutated peptides representing non-self “neo-antigens” exclusively presented on tumor cells. Consequently, such neo-antigens are not affected by central T-cell tolerance and may evoke a more vigorous tumor-selective T cell response [50]. Indeed, mutated epitopes identified in murine melanoma cells have been shown to elicit a stronger T-cell response *in vivo* in a side-by-side comparison with corresponding wild type epitopes [48]. Moreover, the combination of genomics and bioinformatics approaches have revealed that responsiveness to checkpoint blockade immunotherapy appears to be strongly associated with T cells specific for mutant tumour-antigens which are reactivated following treatment with anti checkpoint inhibitors [51,52,53].

Such tumour-specific mutant antigens represent a new frontier of cancer immunotherapy and are natural potential candidates for developing personalized cancer-specific vaccines or could be used for *ex vivo* expansion of patient-derived T cells (TIL or PBMC) before adoptive T cell therapy as well as efficiently targeted with T-cell receptor (TCR) transduced T cells. However, only a small fraction of such mutated peptides are indeed presented by MHC or recognized by T cells, and the number seems to directly correlate with the tumor-specific mutation load [48,54,55,56,57]. Therefore, prediction of MHC presentation calculated by software algorithms need to be confirmed by experimental procedures (e.g. ELISPOT, Tetramer binding assay).

### 5.3. Immune Monitoring 

Vaccine development and vaccination protocols can greatly benefit from high throughput and integrated platforms for immune monitoring of patients administered with cancer vaccines [58].

T cell response to a specific antigen is analyzed by several immune assays including direct multimer staining, ELISPOT, intracellular cytokine staining, and proliferation assays, based on detection following stimulation of the T cells in vitro with antigen [59,60]. However, many of these methods suffer from low sensitivity, and they show a significant variability in the quantitative readouts [61,62,63]. In this framework, next-generation sequencing has recently emerged as a highly sensitive method for characterization of the immune repertoire, assessing individual clonotypes identified based on their unique T cell receptor rearrangements [64,65]. Very recently, next-generation sequencing and immune assays have been combined to identify antigen-specific T cells with a very high sensitivity [66].

Peptide-MHC microarray technologies have been developed for evaluation of T cell reactivity against hundreds to thousands of different MHC complexes per each vaccinated patient [67]. More recently, a cellular microarray assay has been developed with improved sensitivity to identify antigenic T cell specificities in peripheral blood circulating CD8 T cell populations [68]. Furthermore, an antigen-specific T cell phenotyping microarray platform that combines grating-coupled surface plasmon resonance imaging (GCSPRI) and grating-coupled surface plasmon coupled emission (SPCE) fluorescence detection modalities has been shown to be a rapid, highthroughput T cell screening and characterization tool [69].

## 6. Conclusions

Systems vaccinology holds considerable promises for discovery and new insights into processes as complex as innate immunity and the downstream adaptive immune response. Such an integrated approach will have a big impact on vaccine development, providing molecular prediction markers of the immunogenicity of a vaccine, uncovering new correlates of vaccine efficacy as well as guiding the design of new vaccine antigens or formulations. Moreover, such system level approaches could permit the identification of vaccine responders *versus* non-responders, allowing a better immunological coverage of the licensed vaccines. Indeed, gene transcriptional profiling has identified several predictors of immune response to vaccines for infectious diseases. Although a “universal signature” has not been identified yet, shared signatures to vaccines of the same type are likely to be confirmed.

In regards to therapeutic cancer vaccines, vaccinomics is and will be more and more relevant for TAA discovery and validation as well as cellular immune response assessment.

New technical advancements will foster development of ready-to-use chips for easy and rapid screening of vaccinees to improve the outcome of vaccinations. Systems vaccinology represents the real turning point for the switch from the “empirical” to the “knowledge-based” age of the vaccinology, enabling the development of even more successful vaccines for preventive as well as therapeutic intervention strategies for human diseases. In particular, this represents an extraordinary opportunity to improve cancer immunotherapy and clinical outcome in cancer patients on a global scale.

## List of Abbreviations

Ad5 – Adenovirus 5
CRC – colorectal cancer
ELISA – enzyme-linked immunosorbent assay
ELISPOT – Enzyme-Linked ImmunoSpot
GB – glioblastoma
GCSPRI – grating-coupled surface plasmon resonance imaging
HepB – Hepatitis B virus vaccine
HLA – human leukocyte antigen
IFNγ – interferon gamma
MHC – major histocompatibility complex
MMR-II – measles, mumps, and rubella vaccine
MS – Mass-spectrometry
PBMC – peripheral blood mononuclear cells
RCC – renal cell cancer
SNP – single nucleotide polymorphism
SPCE – surface plasmon coupled emission
TAAs – tumor-associated antigens
TCR – T-cell receptor
TLR – Toll-like receptor
TILs – tumor infiltrating lymphocytes
